# Does sleep disruption mediate the effects of childhood maltreatment on brain structure?

**DOI:** 10.1080/20008198.2018.1450594

**Published:** 2018-03-28

**Authors:** Martin H. Teicher, Kyoko Ohashi, Alaptagin Khan, Laura C. Hernandez Garcia, Torsten Klengel, Carl M. Anderson, Marisa M. Silveri

**Affiliations:** a Department of Psychiatry, Harvard Medical School, Boston, MA, USA; b Developmental Biopsychiatry Research Program, McLean Hospital, Belmont, MA, USA; c Neurobiology of Fear Laboratory, McLean Hospital, Belmont, MA, USA; d Brain Imaging Center, McLean Hospital, Belmont, MA, USA; e Neurodevelopmental Laboratory on Addictions and Mental Health, McLean Hospital, BelmontMA, USA

**Keywords:** Abuse and neglect, maltreatment, hippocampus, prefrontal cortex, sleep, actigraphy, depression and anxiety, Abuso y negligencia, maltrato, hipocampo, corteza prefrontal, sueño, actigrafía, depresión y ansiedad, 虐待和忽视, 虐待, 海马, 前额叶, 睡眠, 活动记录仪, 抑郁和焦虑, • Sleep efficiency is disrupted in teenagers who were maltreated as children. • Reduced sleep efficiency is associated with reduced volume of hippocampus, dentate gyrus, insula and inferior frontal cortex. • Reduced sleep efficiency mediates about 45% of the association between maltreatment and hippocampal, dentate gyrus and inferior frontal cortex volume.

## Abstract

**Background**: Childhood maltreatment is associated with alterations in morphology of stress susceptible brain regions. Maltreatment is also known to markedly increase risk for psychopathology and to have an enduring disruptive effect on sleep.

**Objective**: To determine whether abnormalities in sleep continuity have effects on brain morphometry and to evaluate the extent to which sleep impairments mediate the effects of maltreatment on brain structure.

**Method**: Maltreatment and Abuse Chronology of Exposure (MACE) scale ratings, actigraph-assessed sleep and 3T MRI were obtained on *N* = 37 18–19-year-old participants recruited from the community (*N* = 34 with neuroimaging).

**Results**: Fourteen participants had no history of maltreatment while *N* = 23 were exposed, on average, to 4.7 types of maltreatment. Multiplicity of maltreatment was strongly associated with reduced sleep efficiency, increased wake after sleep onset time and number/duration of awakenings, which were independent of effects of maltreatment on depression and anxiety. The most important predictors of impaired sleep were exposure to parental non-verbal emotional abuse at 9–10 years of age. Reduced sleep efficiency correlated with reduced grey matter volume in hippocampus including CA1 subfield, molecular layer and dentate gyrus as well as inferior frontal gyrus and insula. Sleep mediated 39–46% of the effects of maltreatment on volume of hippocampal structures and inferior frontal gyrus.

**Conclusions**: Actigraph-assessed sleep is disrupted in maltreated late teens and mediates a significant portion of the effects of maltreatment on hippocampal volume. Studies are needed to assess whether efforts to enhance sleep in maltreated children can pre-empt or ameliorate neurobiological consequences of maltreatment.

## Antecedentes: el maltrato infantil se asocia con alteraciones en la morfología de regiones cerebrales susceptibles al estrés. También se sabe que el maltrato aumenta notablemente el riesgo de psicopatología y tiene un efecto perturbador permanente sobre el sueño.

**Objetivo:** determinar si las anomalías en la continuidad del sueño tienen efectos sobre la morfometría cerebral y evaluar en qué medida las alteraciones del sueño intervienen en los efectos del maltrato sobre la estructura del cerebro.

**Método:** Se obtuvieron las puntuaciones de la Escala de cronología de la exposición al maltrato y al abuso (MACE, por sus siglas en inglés), sueño evaluado por actigraph y 3T MRI en *N* = 37 participantes de 18-19 años reclutados en la comunidad (*N* = 34 con neuroimagen).

**Resultados:** Catorce participantes no tenían antecedentes de malos tratos mientras que *N* = 23 habían estado expuestos, de media, a 4.7 tipos de maltrato. La multiplicidad de los malos tratos se asoció fuertemente con una menor eficiencia del sueño, tiempo mayor de vigilia después de la hora de inicio del sueño y el número / duración de despertares, que fueron independientes de los efectos del maltrato sobre la depresión y la ansiedad. Los predictores más importantes de problemas de sueño fueron la exposición al abuso emocional no verbal de los padres a los 9-10 años de edad. La reducción de la eficiencia del sueño se correlacionó con la reducción del volumen de la materia gris en el hipocampo, incluido el subcampo CA1, la capa molecular y la circunvolución dentada, así como la circunvolución frontal inferior y la ínsula. El sueño mediaba en el 39-46% de los efectos del maltrato sobre el volumen de las estructuras del hipocampo y la circunvolución frontal inferior.

**Conclusiones:** el sueño evaluado por Actigraph se ve alterado en adolescentes mayores maltratados y media en una parte importante de los efectos del maltrato sobre el volumen del hipocampo. Se necesitan estudios para evaluar si los esfuerzos para mejorar el sueño en los niños maltratados pueden adelantar o mejorar las consecuencias neurobiológicas del maltrato.

## 背景：童年虐待和应激易感脑区的形态变化有关。虐待也被认为可以显著增加心理病态风险和长期干扰睡眠。

**目标：**考察睡眠异常是否对脑部形态测量有影响，以及评估睡眠障碍多大程度中介虐待对脑结构的影响。

**方法：**《长期虐待暴露量表（MACE）》，睡眠活动记录和3T MRI 用于测量37名18到19岁的来自社区的被试。其中34名有神经影响数据。

**结果：**14名被试没有虐待历史，另外23名曾暴露于虐待创伤（平均4.7种虐待）。虐待的多样性显著相关于睡眠效率减少、入睡后更多惊醒和惊喜的数量时间，这种虐待对抑郁和焦虑的效应是独立的。对睡眠损害的最主要的预测因子是“在9-10岁暴露于家长的非言语性情绪虐待”。睡眠效率减少和海马（包括 CA1子区、分子层和齿状回和）、额下回和脑岛的灰质容量相关。睡眠中介了虐待经历对海马结构和额下回容量的效应的39-46%。

**结论：**青少年晚期的虐待经历干扰了由活动记录仪评估的睡眠质量，同时睡眠显著中介了虐待对海马容量的影响效应。还需要更多研究来评估，是否提高受虐待儿童的睡眠可以预防或者减轻虐待带来的神经生理改变。

## 1. Background

Childhood maltreatment is the most important preventable risk factor for psychopathology. Adverse childhood experiences in the form of maltreatment and household dysfunction have been reported to account for 45, 54 and 67% of the population attributable risk for childhood-onset psychiatric disorders (Green et al., 2010), depression (Dube, Felitti, Dong, Giles, & Anda, 2003) and suicide attempts (Dube et al., 2001), respectively. It is critically important to understand how maltreatment can increase risk in order to develop more effective strategies to enhance resilience, and to pre-empt or treat adverse outcomes. We are defining maltreatment, following the World Health Organization, as all forms of physical and/or emotional ill-treatment, sexual abuse, neglect or negligent treatment resulting in actual or potential harm to the child’s health, survival, development or dignity in the context of a relationship of responsibility, trust or power.

**Figure 1.**
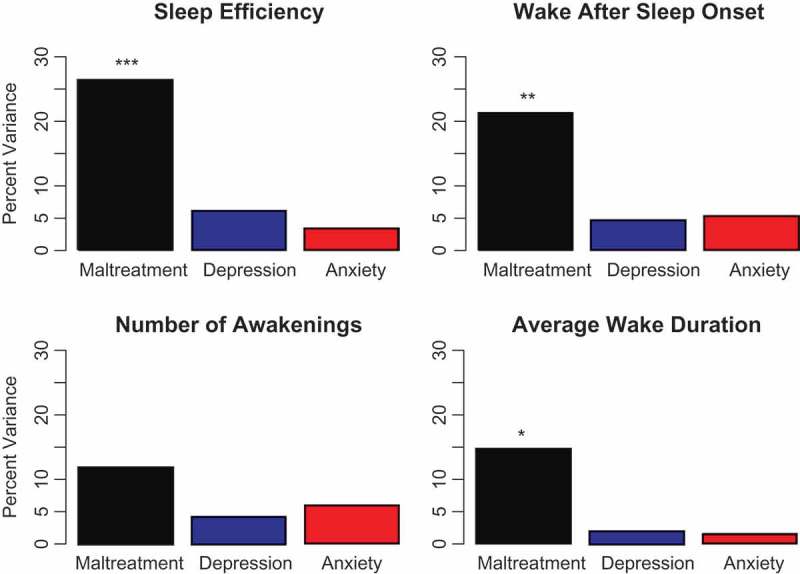
Multiple regression analyses with variance decomposition indicating the percent variance in actigraph-assessed sleep measures accounted for by number of types of maltreatment and current symptoms of depression and anxiety. *p < .05, **p < .02, ***p < .001.

**Figure 2.**
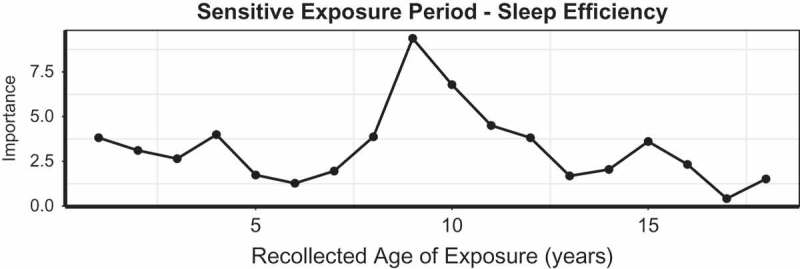
Random forest regression with conditional trees indicating the importance of parental non-verbal emotional abuse as a predictor of sleep efficiency during each year of childhood.

A growing body of reproducible findings, from our lab and others, links maltreatment to brain differences. The most consistent findings are smaller midsagittal area or decreased fractional anisotropy of the corpus callosum and lower hippocampal volume in adults but not necessarily in youths (see Teicher and Samson, 2016; Teicher, Samson, Anderson, and Ohashi, 2016, for recent reviews). Maltreatment is also associated with attenuated development of the anterior cingulate, orbitofrontal and dorsolateral prefrontal cortex, and with enhanced amygdala response to threatening stimuli and reduced striatal response to reward (Teicher & Samson, 2016; Teicher et al., 2016).

Several years ago we developed a cascade model to explain the association between maltreatment, brain development and psychopathology (Teicher, Andersen, Polcari, Anderson, & Navalta, 2002). Briefly, we proposed that maltreatment or early life stress produces a cascade of events. The first stage involved the programming of stress response systems to augment stress responses. These neurohumors subsequently produce effects on neurogenesis, synaptic overproduction and pruning, and myelination leading to alterations in the previously mentioned regions. These alterations, in turn, comprise a neurobiological framework through which maltreatment increases risk of developing psychopathology.

We now recognize that programming of stress responses likely occurs through epigenetic modifications, such as DNA methylation (DNAm), within genes involved in cortisol regulation. This is consistent with the Carrion, Weems, and Reiss (2007) observation in a pilot longitudinal study that PTSD symptoms and baseline cortisol levels predicted hippocampal volume levels over an ensuing 12–18 month period, and also with their cross-sectional finding of a significant inverse correlation between pre-bedtime cortisol and volume of left ventral prefrontal cortex in youths with PTSD symptoms (Carrion, Weems, Richert, Hoffman, & Reiss, 2010). It is also highly concordant with finding from Klengel et al. (2013) of an allele-specific effect of childhood maltreatment on DNAm of elements of the FKBP5 gene leading to increased stress-dependent gene transcription, long-term dysregulation of cortisol secretion and effects on hippocampal morphometry.

However, in addition to epigenetically-mediated effects of childhood maltreatment on stress hormone levels, it also conceivable that maltreatment may be affecting trajectories of brain development by disrupting sleep given the important role that sleep plays in brain development. Objective assessment of sleep using ambulatory activity monitors (actigraphs) show that maltreated children have sleep disruptions most consistently reflected in reduced sleep efficiency, and that this was more strongly associated with degree of exposure to physical abuse than sexual abuse or PTSD (Glod, Teicher, Hartman, & Harakal, 1997; Sadeh et al., 1995). Sleep impairment is a highly enduring effect of maltreatment, quite discernible even 10 years after disclosure of substantiated events (Noll, Trickett, Susman, & Putnam, 2006).

Sleep appears to be an obligatory process in animals and many cognitive functions are disrupted if we do not sleep. The synaptic homeostasis hypothesis posits that sleep is the currency the brain pays for plasticity, to consolidate what we have learned, and to be ready to learn new things (Tononi & Cirelli, 2016). Sleep accomplishes this by exerting an essential housekeeping role in the maintenance of synaptic function by decreasing synaptic strength and helping to remove excessively utilized and damaged synapses through astrocytic phagocytosis (Bellesi et al., 2017). Preclinical studies show that extended sleep disruption in adolescence affects cellular structure of prefrontal cortical pyramidal cells (De Vivo et al., 2016) and leads to a state of sustained microglia activation that can increase the brain’s susceptibility to other forms of damage (Bellesi et al., 2017). Several studies have also shown that sleep deprivation impairs hippocampal synaptic plasticity (Prince & Abel, 2013). Clinical research assessing the acute effects of sleep loss in human children report effects on network architecture and myelination (Kurth et al., 2016).

## 2. Objective

The aim of this study was to assess the relationship between sleep continuity and morphometry of stress-susceptible brain regions and to determine whether sleep impairment mediates effects of maltreatment on these structures in a cross-sectional sample of 18–19-year-olds, including several participants with moderate-to-high exposure to childhood maltreatment.

## 3. Methods

### 3.1. Participants

The McLean Hospital Institutional Review board approved this study. Participants provided written informed consent after the study was fully explained and questions answered. They were screened, recruited and evaluated using previously published methods (Khan et al., 2015; Teicher, Anderson, Ohashi, & Polcari, 2014). Recruited participants were medically healthy, right handed, 18–19 years of age, and unmedicated with the exception of oral contraceptives, hormone replacement therapy or occasional use of non-steroidal asthma inhalers or non-sedating antihistamines. Participants were selected based on exposure history and not psychopathology, except that high levels of drugs and alcohol use (e.g. more than 15 days per month, or any use of cocaine or heroin) were grounds for exclusion. Participants were paid US$25 for completing online assessments, US$100 per interview and assessment session (typically one 4-hour session) and US$100 for a one hour MRI protocol.

The sample consisted of 37 (13 male/24 female) unmedicated, right-handed individuals. Data on ethnicity, education, parental education, family income and perceived financial sufficiency during childhood (rated 1 = much less than enough money to meet our needs to 5 = much more than enough money to meet our needs) were collected. We included perceived financial sufficiency as an alternative to family income, as participants were often uncertain of their parents’ income, and family income could mean very different things depending on locale, family size and parental spending habits. General demographic information is presented in Table 1.

### 3.2. Assessments

#### 3.2.1. Maltreatment measures: self report

Type and timing of exposure to maltreatment was assessed using the Maltreatment and Abuse Chronology of Exposure (MACE) scale, which was specifically developed to test hypotheses about sensitive periods by assessing severity of recollected exposure to maltreatment, including peer victimization, during each of the 18 years of childhood (Teicher & Parigger, 2015). The scale was developed using item response theory and provides excellent overall test-retest reliability (Teicher & Parigger, 2015). Participants indicate whether they experienced a given event and check off each year of occurrence to provide ratings on exposure to 10 types of MAL across childhood. MACE scores are determined by the numbers of different items of increasing severity endorsed within each maltreatment category and are not affected by the number of times that the events occurred.

**Sexual abuse** was indicated by parents or other adults acting in a sexually inappropriate manner including sexual comments, touching or foundling ‘you’ in sexual way, having ‘you’ touch them in sexual way, or intercourse, or by peers forcing ‘you’ to do things sexually that ‘you’ did not want to do, or forcing ‘you’ to engage in sexual activities against your will. **Parental verbal abuse** was characterized by parents swearing at ‘you’, calling ‘you’ names or insulting ‘you’, saying things that made ‘you’ feel humiliated saying things that made ‘you’ feel physically threatened, or threatening to leave or abandon ‘you’. **Non-verbal emotional abuse** (NVEA) was characterized by parents being very difficult to please, having no time or interest in talking to ‘you’, having ‘you’ shoulder adult responsibilities at inappropriate ages, keeping important facts or secrets from ‘you’, or locking ‘you’ in closet, garage, basement, etc. **Parental physical maltreatment** was indicated by various forms of spanking (e.g. unclothed bottom; use of paddle or belt), intentionally pushing, pinching, slapping, or kicking ‘you’, hitting so hard as to leave marks, or to require medical attention. **Witnessing interparental violence** was indicated by seeing adults living in household push, slap, or throw something at mother (stepmother, grandmother) or father (stepfather, grandfather), hitting so hard as to leave marks or to require medical attention. **Witnessing sibling abuse** was characterized by seeing parents or adults living in household hit your sibling (stepsibling) so hard that it left marks or require medical attention, making inappropriate sexual comments or touching or fondling sibling in a sexual way. **Peer emotional abuse** was indicated by peers swearing at ‘you’, calling ‘you’ names or insulting ‘you’, saying things that made ‘you’ feel humiliated, saying things behind your back, spreading rumours, posting derogatory information, excluding ‘you’ from groups/activities or acting in a threatening manner. **Peer physical abuse** was characterized by intentionally pushing, shoving, or kicking ‘you’, hitting ‘you’ so hard as to leave marks or require medical attention, physically threatening ‘you’ in order to take your money or possessions, or forcing ‘you’ to do things ‘you’ did not want to do. **Emotional neglect** was indicated by maternal and paternal emotional unavailability for reasons such as drugs, alcohol, being a workaholic, having an affair, heedlessly pursuing their own goals, and failure of family members to make ‘you’ feel loved, help ‘you’ feel special/important or help serve as a source of strength or support. **Physical neglect** was characterized by having to wear dirty clothes, not having enough to eat or absence of family members there to take care of and protect ‘you’, take ‘you’ to doctors or ER if need were to arise, or to look out for ‘you’.

Total MACE scores correlated 0.738 and 0.698 with childhood trauma questionnaire (CTQ) and the Adverse Childhood Experience (ACE) scores, but accounted for 2.00- and 2.07-fold more of the variance in psychiatric symptom ratings (*n* = 1051) based on variance decomposition. A key advantage of the MACE is that each type of exposure fits a Rasch Model meaning that it provides a fundamental measurement of exposure in which items are measured on an interval scale with a common unit (Bond & Fox, 2007).

#### 3.2.2. Maltreatment measures: interview

Semi-structured Traumatic Antecedents Interviews (100-items) (Herman, Perry, & Van Der Kolk, 1989) were also conducted on each participant to gauge consistency and to verify reliability of MACE responses.

#### 3.2.3. Diagnoses

Structured Clinical Interviews for DSM-IV Axis I and II psychiatric disorders (First, Spitzer, Gibbon, & Williams, 1997) were used to establish current and lifetime diagnoses.

#### 3.2.4. Current symptom ratings

Kellner’s Symptom Questionnaire (Kellner, 1987) was used to provide measures of current severity of depression, anxiety somatization and anger-hostility. The Symptom Questionnaire consists of 92 yes/no or true/false items and assesses both well-being and pathology. This makes the scale highly sensitive to change and capable of detecting low levels of symptomatology, such as the difference between healthy controls and euthymic individuals with bipolar disorder (Kellner, 1987).

#### 3.2.5. Actigraphy and sleep analyses

Rest–activity data were collected for seven days using ActiGraph GT9X Link (ActiGraph, Pensacola, FL), which detects movement (accelerations) of the non-dominant wrist using triaxial accelerometers in 10-s epochs. Each participant’s time series was analysed using the Cole-Kripke algorithm (Cole, Kripke, Gruen, Mullaney, & Gillin, 1992) implemented in the ActiLife 6 software. The software provided measures of sleep efficiency, wake after sleep onset, number of awakenings and duration of awakenings. Sleep latency was not determined as the start of the sleep period was set to coincide with actigraph-determined onset. Sleep efficiency was calculated as percent time spent asleep from sleep onset to final awakening. Prior studies have shown that the Cole-Kripke algorithm correctly distinguished sleep from wakefulness approximately 88% of the time, and that actigraphic sleep percentage and sleep latency estimates correlated 0.82 and 0.90, respectively, with corresponding polysomnography measures. Excellent between-day intraclass correlation coefficient of 0.77 was observed using this device, to assess sleep efficiency in 25 separate participants across seven monitoring days (unpublished data).

### 3.3. Image acquisition

High-resolution T1-weighted MRI datasets were acquired on 34 (13 male/21 female) participants using a TIM Trio Scanner (3T; Siemens AG, Siemens Medical Solutions, Erlangen, Germany) with a 32-element phased-array RF reception coil. Scan parameters were: the sagittal plane, TE/TR/TI/fli*p =* 2.74 ms/2.1 s/1.1 s/12 deg; 3D matrix 256 × 256 x 128 on 256 × 256 x 170 mm field of view; bandwidth 48.6 kHz; GRAPPA factor of 2 and scan time 4:56.

### 3.4. Image analyses

Volumetric segmentation was performed with the FreeSurfer image analysis suite Version 6.0, Technical details of these procedures are described in prior publications (Dale, Fischl, & Sereno, 1999; Fischl et al., 2004) including refined software for segmentation of the hippocampus (Iglesias et al., 2015). Overall, this approach provides hippocampal subfield volume measures that more closely align with histological measurements compared to the prior FreeSurfer release or alternative automated segmentation algorithms (Iglesias et al., 2015).

### 3.5. Statistical analyses

#### 3.5.1. Effects of maltreatment

Statistical associations between number of types of maltreatment and actigraph measures of sleep and MRI measures of brain morphometry were assessed using bivariate regression with correction for multiple comparisons (Benjamini & Hochberg, 1995; Hochberg & Benjamini, 1990) when evaluating multiple independent outcomes. Multiple regression analyses were used to assess the effects of maltreatment on sleep measures controlling for potential mediating effects of anxiety and depression. Percent variance attributable to maltreatment versus depression and anxiety ratings was estimated using variance decomposition analyses which takes into account cross-correlations between the predictor variables (Grömping, 2007; Lindeman, Merenda, & Gold, 1980). Statistical analyses were conducted in R (version 3.3.3) using packages ‘relaimpo’, ‘party’ and ‘mediation’.

#### 3.5.2. Importance of type of maltreatment on sleep: random forest regression with conditional trees

A potential problem in identifying importance of type and timing of exposure to maltreatment is collinearity as there is generally a strong correlation between degree of exposure to maltreatment at adjacent ages or between different types of abuse. This problem can markedly interfere with the interpretation of results using multiple regression analysis or structural equation modelling. An alternative approach is to use predictive analytical techniques with artificial intelligence algorithms such as random forest regression with conditional inference trees (Breiman, 2001; Liaw & Wiener, 2002; Svetnik et al., 2003). This modern computationally demanding technique is well suited to the analysis of highly collinear data sets and can handle models with a very large number of predictor variables and does not assume a linear relationship between predictor variables and outcome (Breiman, 2001; Liaw & Wiener, 2002; Svetnik et al., 2003). We found using Monte-Carlo simulations with actual exposure data (*N* = 530) and simulated sensitive period outcomes that random forest regression with conditional trees (Strobl, Boulesteix, Zeileis, & Hothorn, 2007) most accurately identified the type and timing of exposure used to generate the outcomes (compared to conventional random forest, gradient boosted machines, support vector machines, neural networks or generalized linear models), and have used this approach in recent reports (Khan et al., 2015; Schalinski & Teicher, 2015; Schalinski et al., 2016).

Our primary interest was to identify types of maltreatment that were most important predictors of disturbed sleep, and then to identify ages when exposure to this type of maltreatment was most predictive. Training and testing were accomplished using 100 repetitions of leave group out cross validation (75% training/25% testing; Kuhn, 2008), which were averaged to evaluate the importance of each of the predictors to the outcome. Importance was assessed by sequentially permuting each of the predictor variables to ascertain how much this degraded the accuracy of the predicted fit, as indicated by the increase in mean square error. Permuting important predictors produces a large increase in mean square error while permuting unimportant predictors has a negligible effect.

To assess the significance of these mean importance measures the same analyses were re-run 5000 times using reshuffled volume measures to obtain the probability that importance of each predictor could have occurred by chance. This procedure corrects for multiple comparisons and provides more conservative estimators of significance than parametric tests with Bonferroni correction.

#### 3.5.3. Causal mediation analysis

Causal mediation was assessed using a model based approach and the sequential ignorability assumption to derive point estimates following the work of Imai et al. (Imai, Keele, & Tingley, 2010). Linear mediator and outcome models were used as inputs to the mediate function (R package ‘mediation’), which computes the estimated average causal mediation effect and other quantities of interest. Estimated parameters were obtained from 2000 stimulations using a quasi-Bayesian Monte Carlo method (Tingley, Yamamoto, Hirose, Keele, & Imai, 2014).

## 4. Results

### 4.1. Types of exposure and clinical characteristics

The sample consisted of 14 individuals with no self-reported exposure to childhood maltreatment and 23 individuals reporting, on average, exposure to 4.7 ± 2.1 different types of maltreatment on the MACE. As indicated in Table 1, the majority of participants reported exposure to parental verbal abuse, parental NVEA, peer emotional abuse and emotional neglect. Approximately one-third of the sample reported exposure to each of the other types of maltreatment.

**Table 1.** Participant’s general demographic information and types of maltreatment experienced by maltreated participants

Age (years)	18.8 ± 0.5	Types of maltreatment	Prevalence (%)
Participant education (years)	12.7 ± 0.8	Sexual Abuse	35
Parental education (years)	16.7 ± 3.8	Parental Verbal Abuse	65
Financial sufficiency during childhood		Parental Non-Verbal Emotional	57
Much less than enough money	8.1%	Parental Physical Abuse	39
Less than enough money	18.9%	Witness Interparental Violence	39
Enough money	35.1%	Witness Violence to Sibs	35
More than enough money	32.4%	Peer Emotional Abuse	65
Much more than enough money	5.4%	Peer Physical Abuse	43
US Census Categories		Emotional Neglect	61
White	70.3%	Physical Neglect	30
Asian	5.4%		
Black	13.5%		
American Indian/Alaska Native/Hawaiian	5.4%		
Other	5.4%		
Hispanic Ethnicity	16.2%		

The most prevalent lifetime diagnoses in the sample were major depressive disorder, social phobia, specific phobia, ADHD, generalized anxiety and panic disorder (Table 2). The most prevalent personality disorders were avoidant, depressive, dependent and paranoid. PTSD was rare, with only one maltreated participant meeting full criteria and three participants meeting partial criteria. Current symptoms of depression, anxiety, somatization and anger-hostility were markedly higher in maltreated than non-maltreated participants, despite enrolling both groups based on exposure history without regard to presence or absence of psychopathology. Cohen effect size differences were large and ranged from 1.02 (somatization) to 1.8 (depression). There were particularly strong associations between multiplicity of exposure to maltreatment and self-report ratings of depression (F_1,35_ = 23.84, *p =* .00003) and anxiety (F_1,35_ = 16.63, *p =* .00025).

**Table 2.** Lifetime psychiatric history and current symptom scores for participants

Measures	Non-maltreated	Maltreated
*Lifetime History SCID*	*Prevalence*	*Prevalence*
Major Depression	21%	61%
Any Anxiety Disorder	21%	78%
Social Phobia	7%	48%
Special Phobia	7%	39%
Generalized Anxiety	7%	22%
Panic Disorder	0%	22%
PTSD (full)	0%	4%
PTSD (partial)	0%	13%
ADHD	7%	35%
Eating Disorder	0%	13%
Any personality disorder	0%	43%
Avoidant	0%	22%
Depressive	0%	13%
Dependent	0%	9%
Paranoid	0%	9%
Passive Aggressive	0%	4%
Obsessive Compulsive	0%	4%
Histrionic	0%	4%
Schizoid	0%	4%
Borderline or Narcissistic	0%	0%
Schizotypal or Antisocial	0%	0%
*Current Psychiatric Symptoms*	*Mean ± SD*	*Mean ± SD*
Anxiety (SQ)	3.50 ± 3.76	11.13 ± 5.50^†^
Depression (SQ)	2.29 ± 3.32	10.08 ± 5.13^¥^
Somatization (SQ)	4.04 ± 4.17	9.80 ± 6.77*
Anger-Hostility (SQ)	3.38 ± 2.34	7.81 ± 4.16**

*p < .01, **p < .001, ^†^ *p *< .0001, ^¥^ *p *< .00002; *p* values and means from ANCOVA with age, gender, financial sufficiency and parental education as covariates.

SCID = Structured Clinical Interview for DSM-IV disorders, SQ = Kellner Symptom Questionnaire.

### 4.2. Maltreatment and sleep

As seen in Table 3, maltreated participants tended to have poorer sleep, with bivariate regression analyses indicating significant associations between number of types of maltreatment reported and measures of sleep efficiency, wake after sleep onset (WASO), number of awakenings and average wake duration. Effect sizes for effects of maltreatment on sleep efficiency and WASO were large, while effect sizes for number of awakenings and wake duration were moderate. Effects of maltreatment on sleep measures did not vary significantly by gender.

**Table 3.** Group differences and bivariate associations between numbers of types of maltreatment reported and sleep measures

	Groups*		Regression		
Measures	Non-maltreated	Maltreated	B	r	*p*-value
Sleep Efficiency (%)	89.58 ± 4.00	86.91 ± 4.10	−0.906	.609	.00007
Total Sleep Time (min)	387.6 ± 122.3	359.8 ± 79.8	−7.209	.242	.19
Wake After Sleep Onset (min)	42.42 ± 15.70	51.45 ± 18.19	3.292	.552	.0013
Number of Awakenings	16.98 ± 5.67	20.29 ± 7.00	1.030	.447	.012
Average Wake Duration (min)	2.46 ± 0.45	2.38 ± 0.51	.061	.417	.020

*Means from ANCOVA with covariates for gender, age, financial sufficiency and parental education. B = unstandardized regression coefficient.

A key concern is whether there is a direct association between maltreatment and sleep parameters or if these sleep abnormalities are a consequence of ensuing symptoms of depression and anxiety, given the association between symptoms of depression and anxiety and sleep (e.g. depression and sleep efficiency: *r =* -.356, *p =* .04; anxiety and WASO: *r =* .374, *p =* .04). To address this question we used multiple regression analysis and variance decomposition (Grömping, 2007; Lindeman et al., 1980) to estimate the percent of variance in sleep parameters accounted for by maltreatment per se versus the percentage accounted for by symptoms of depression or anxiety.

As seen in Figure 1, the major share of the variance in these sleep measures was predicted by number of types of maltreatment, which remained a significant predictor even after controlling for symptoms of depression and anxiety. In contrast, current symptoms of anxiety and depression did not significantly predict sleep parameters once maltreatment was accounted for.

The MACE provides information on degree of exposure to 10 types of maltreatment across each year of childhood. While the sample size is too small to justify complete sensitive exposure period analyses (Khan et al., 2015), we sought to ascertain what types of maltreatment best predicted alterations in sleep efficiency, and recollected ages when exposure to this type of maltreatment were most important. Table 4 shows the results of the random forest regression analysis with conditional trees. The most important type of exposure was parental NVEA, followed by parental physical abuse, physical neglect and sexual abuse.

**Table 4.** Random forest regression with conditional trees indicating the importance of exposure to each type of maltreatment assessed by the MACE across childhood

Predictors	Random Forest Regression	
	Importance	Permuted *p*-value
**Sexual Abuse**	**3.90**	.**047**
Parental Verbal Abuse	1.01	.176
**Parental Non-Verbal Emotional Abuse**	**20.70**	.**0006**
**Parental Physical Abuse**	**5.06**	.**045**
Witness Interparental Violence	−0.26	.445
Witness Violence to Siblings	1.34	.094
Peer Emotional Abuse	0.36	.275
Peer Physical Abuse	0.31	.243
Emotional Neglect	3.06	.074
**Physical Neglect**	**4.08**	.**045**

Importance is defined as the increase in mean square error resulting from permuting each predictor variable.

Having identified NVEA as the strongest predictors of sleep efficiency, we used random forest regression with conditional trees to identify specific ages when exposure to NVEA was most predictive. As seen in Figure 2, recollected recall of NVEA at ages nine (*p = *.0006) and 10 (*p =* .0034) were the most important predictors.

### 4.3. Region of interest volume analyses

Based on preclinical studies, planned comparisons were conducted to investigate effects of reduced sleep efficiency on portions of prefrontal cortex, hippocampus and amygdala, corrected for multiple comparisons. Exploratory analyses also were conducted to evaluate effects of reduced sleep efficiency on regions consistently reported to be affected by maltreatment (Teicher et al., 2016) and corrected for total number of comparisons made – both planned and exploratory. As seen in Table 5, there were direct associations between sleep efficiency and grey matter volume (GMV) in hippocampus, particularly CA1 subfield, molecular layer and dentate gyrus, as well as the inferior frontal cortex and insula. There were potential direct associations of sleep efficiency with amygdala, superior frontal cortex, occipital cortex, putamen and cerebellum that did not survive corrections for multiple comparisons. Bivariate analyses were used as there were no significant associations between intracranial volume (ICV) and multiplicity of maltreatment (F_1,31_ = 1.34, *p =* 0.26) or between ICV and sleep efficiency (F_1,31_ = 0.43, *p =* 0.52). There were also no significant interactions between sex and sleep efficiency on GMV with the exception of the insula (F_1,30_ = 5.01, *p =* .03), where effects of sleep efficiency were significant in females but not males.

A key consideration is whether alterations in sleep efficiency mediated the effect of maltreatment on GMV in regions where sleep efficiency emerged as a significant predictor. As seen in Table 6, sleep efficiency mediated 38.5–46.3% of the total effect of maltreatment on hippocampus including CA1 subfield, dentate gyrus and molecular layer and 45.5% of the total effect of maltreatment on the inferior frontal cortex (*p =* .054), but did not mediate a significant portion of the effect of maltreatment on the insula. We also assessed whether sleep disruption was a mediator of psychopathology, but found that sleep efficiency did not mediate a significant portion of the association between number of types of maltreatment and symptoms of anxiety (*p* > .60), depression (*p* > .80), somatization (*p* > .80) or anger-hostility (*p* > .40).

**Table 5.** Bivariate associations between sleep efficiency and grey matter volume of cortical, subcortical and cerebellar regions as well as corpus callosum

Measures	b	*r*	*p*-value	Adj *p*-value*
***Planned Comparisons***				
Amygdala	38.826	.353	.041	.062
**Whole Hippocampus**	**95.446**	.**539**	.**001**	.**003**
**CA1**	**19.197**	.**509**	.**002**	.**005**
**CA3**	**5.013**	.**387**	.**024**	.**044**
**Dentate gyrus**	**9.062**	.**538**	.**001**	.**003**
**Molecular layer**	**16.660**	.**564**	.**001**	.**003**
Anterior Cingulate	85.829	.281	.107	
**Inferior Frontal Cortex**	**221.356**	.**539**	.**001**	**.003**
Middle Frontal Cortex	356.227	.330	.057	
Superior Frontal Cortex	396.477	.346	.045	.062
Orbitofrontal Cortex	44.946	.222	.207	
***Exploratory Analyses***				
**Insula**	**171.579**	.**456**	.**007**	.**029**
Accumbens	0.325	.011	.950	
Caudate	21.253	.119	.503	
Putamen	136.110	.411	.016	.059
Inferior Temporal Cortex	133.605	.388	.023	.062
Middle Temporal Cortex	61.007	.269	.124	
Superior Temporal Cortex	12.712	.093	.601	
Precuneus	52.842	.291	.095	
Occipital Cortex	155.150	.379	.027	.064
Cerebellar Cortex	1116.295	.397	.020	.062
Corpus Callosum Anterior	5.513	.206	.242	
Corpus Callosum Mid Anterior	0.580	.016	.930	
Corpus Callosum Central	7.199	.188	.286	
Corpus Callosum Mid Central	5.977	.243	.166	
Corpus Callosum Posterior	3.191	.100	.575	

Probability values adjusted using method of Benjamini and Hochberg (Benjamini & Hochberg, 1995; Hochberg & Benjamini, 1990) based on number of planned comparisons or total number of comparisons (exploratory analyses).

**Table 6.** Mediation analysis indicating average causal mediation effect of sleep efficiency on relationship between exposure to maltreatment (number of different types) and regional grey matter volume

	Average Causal Mediation Effect		Average Direct Effect		Total Effect		Proportion Mediated	
Regions	Estimate	*p*-value	Estimate	*p*-value	Estimate	*p*-value	Estimate	*p*-value
Hippocampus	−61.92	.043	−68.43	.222	−130.35	.004	0.463	.047
CA1	−10.83	.032	−17.18	.063	−28.01	.003	0.385	.035
Dentate gyrus	−5.49	.022	−7.45	.105	−12.94	.002	0.411	.024
Molecular layer	−10.35	.008	−12.63	.104	−22.98	.002	0.444	.01
Inferior Frontal Cortex	−140.84	.054	−162.82	.171	−303.66	<.0005	0.455	.054
Insula	−80.97	.459	−194.55	.214	−275.52	.002	0.299	.461

The Average Causal Mediation Effect indicates the degree of reduction in grey matter volume attributed to the mediator.

## 5. Discussion

Actigraph indices of sleep continuity were significantly affected by degree of exposure to maltreatment in study participants. These findings are consistent with prior reports showing alterations in actigraph-assessed sleep measures in psychiatrically hospitalized maltreated children (Glod et al., 1997; Sadeh et al., 1995) and in adult outpatients (Schafer & Bader, 2013). Hence, these results extend prior findings to include maltreated individuals living in the community and not currently in psychiatric treatment. Further, maltreatment was found to have a significant effect on sleep efficiency, WASO and duration of awakenings independent of the effects of maltreatment on symptoms of anxiety or depression. This is an interesting and somewhat unexpected finding as impaired sleep is part of the diagnostic criteria for major depression (MDD) and generalized anxiety disorder (GAD).

One concern is whether failure to detect an association between depression or anxiety and sleep measures after correcting for maltreatment was due to insufficient symptom severity (floor effect). This was not the case, as maltreatment was associated with large effect size increases in depression and anxiety, and these symptoms correlated significantly with sleep measures when maltreatment was excluded from the analysis. Another concern is whether impaired sleep was related to PTSD rather than anxiety or depression, however this was not the case, as only one participant met lifetime diagnostic criteria for PTSD and only three participants met partial criteria. This is not surprising as PTSD is only the fifth (Ackerman, Newton, McPherson, Jones, & Dykman, 1998) or tenth (Copeland, Keeler, Angold, & Costello, 2007) most common diagnosis in childhood, following exposure to traumatic stressors. Further, participants in this sample were more likely to have experienced emotional neglect or abuse than types of maltreatment considered traumatic.

We have advanced the hypothesis that psychiatric disorders can be subdivided based on presence or absence of history of maltreatment, with the maltreated ‘ecophenotype’ typically having an earlier age of onset, more severe course, greater number of comorbid diagnoses and poorer response to treatment (Teicher & Samson, 2013). Further, the maltreated ecophenotype has morphological and functional brain abnormalities and alterations in inflammatory markers not present in non-maltreated individuals with the same primary diagnosis (Teicher & Samson, 2013). These present findings lead us to wonder whether sleep impairments are more characteristic of the ecophenotype and if they are actually comorbid consequences of maltreatment rather than intrinsic components of the diagnosis. A direct answer to this question will require studies with maltreated and non-maltreated individuals with GAD or MDD as well as maltreated individuals without psychopathology.

Parental NVEA was the most important predictor of sleep efficiency with recollected exposure at 9–10 years of age being particularly important. This is consistent with the prior report of Schafer and Bader (2013), who found that actigraph-assessed sleep in adult outpatients was affected by stress load prior to age 13, but not associated with stress during adolescence or adulthood. Parental NVEA emerged as a discrete form of emotional abuse during construction of the MACE (Teicher & Parigger, 2015). We found using item response theory that certain types of emotional abuse (e.g. parent being hard to please, parent having no interest in spending time with you, parents keeping important secrets from you) did not load onto the same latent trait as more verbal aspects of emotional abuse. We subsequently found that parental NVEA at age 14 was the most important predictor of risk for developing MDD and current symptoms of MDD in males (Khan et al., 2015). It is remarkable that NVEA at 9–10 years may be the most important predictor of sleep efficiency a decade later, and this association will need to be confirmed with longitudinal studies.

Reduced sleep efficiency was the sleep parameter most strongly associated with maltreatment, and sleep efficiency was a significant predictor of GMV in hippocampus (including CA1, molecular layer and dentate gyrus) as well as inferior frontal cortex and insula. These associations emerged from planned comparisons of regions reportedly affected by sleep loss and an exploratory analysis of regions of interest previously identified as affected by maltreatment (Teicher & Samson, 2016; Teicher et al., 2016) and corrected for multiple comparisons. Further, sleep efficiency was found to mediate the association between maltreatment and measures of whole hippocampal and hippocampal subfield volumes, and volume of inferior frontal cortex.

Actigraphy has both strengths and weaknesses as a means of assessing sleep. It is more objective and often more reliable than sleep diaries or rating scales (Kaplan, Talbot, Gruber, & Harvey, 2012; Werner, Molinari, Guyer, & Jenni, 2008) and, in comparison to polysomnography, can be conveniently acquired over several days with subjects sleeping in their own beds without intrusive electrodes (Kaplan et al., 2012). Actigraphic estimates of sleep versus wake state correlate reasonably well with polysomnography, though this depends on age, algorithms and actigraph settings (Meltzer, Walsh, Traylor, & Westin, 2012). As Tryon (2004) indicates, sleep onset is a gradual process and actigraphy identifies an earlier phase of the sleep-onset process than polysomnography, which results in systematic differences. The critical limitation of actigraphy and sleep diaries is that they only provide information on sleep–wake while polysomnography provides detailed information on sleep architecture. This is quite important as the restorative effects of sleep appear to depend on the amount of slow wave sleep and REM (Diekelmann & Born, 2010). Hence, although we observed significant associations between reduced sleep efficiency and regional GMV, direct associations may stem from maltreatment-related effects on stage III or IV slow-wave sleep or REM.

Additional limitations of the current study include modest sample size and reliance on retrospective cross-sectional analyses and self-report. Modest sample size may have precluded detection of more subtle effects of impaired sleep efficiency on brain structures, including several associations in Table 5 with *p* values between .059 and .064 after correction for multiple comparisons. Concerns about the veracity of self-report stem largely from the recovered memory debate (Berger, 2002; Brown, 1995; Porter, Yuille, & Lehman, 1999), which does not deny that abuse occurs but disputes the capacity of individuals to fully repress then recover seminal memories as they should be profound and enduring. This is not relevant in this case, as all participants had sustained memories of their abuse. Additional criticisms include memory impairment associated with psychopathology, and mood-congruent memory biases. Brewin, Andrews, and Gotlib (1993), in a comprehensive review, found scant evidence to support these criticisms. Modern instruments such as the CTQ (Bernstein & Fink, 1998) and MACE (Teicher & Parigger, 2015) focus on the occurrence of events rather than attributions and do not ask whether individuals believed they were abused, but whether or not they experienced particular events. When presented in this way these tests have impressive long-term test-retest reliability (e.g. CTQ 0.88 [Bernstein & Fink, 1998], Childhood Abuse and Trauma Scale, *r =* .89 [Sanders & Becker-Lausen, 1995], MACE *r =* .91 [Teicher & Parigger, 2015]) and absence of a significant negative attribution bias (Teicher & Parigger, 2015). In psychiatric studies we frequently rely on subject’s recollection, and it is hard to think of anything that we ask subjects to recollect with significantly higher test-retest reliability than maltreatment scores. Neurobiology also provides remarkable convergent support, as unbiased whole brain analyses delineate alteration specifically in visual cortex (Tomoda, Polcari, Anderson, & Teicher, 2012) and visual-limbic pathway (Choi, Jeong, Polcari, Rohan, & Teicher, 2012) in adults visually witnessing domestic violence, in auditory cortex (Tomoda et al., 2011) and auditory pathway (Choi, Jeong, Rohan, Polcari, & Teicher, 2009) in adults reporting parental verbal abuse, and thinning of the genital representation area of the somatosensory cortex in women reporting childhood sexual abuse (Heim, Mayberg, Mletzko, Nemeroff, & Pruessner, 2013). In short, retrospective reports are much more reliable and verifiable than critics are generally aware. There remains, however, a compelling need for longitudinal studies to delineate causal relationships, which cannot be determined through retrospective cross-sectional analyses.

Finally, this study raises a number of questions of potential clinical importance. First, would improving the sleep quality of 18–19-year-old maltreated individuals with reduced hippocampal volume result in some recovery of hippocampal volume? This is possible as the hippocampus is remarkably plastic (Davidson & McEwen, 2012). Second, would monitoring and improving sleep quality in maltreated children, once abuse became evident, have a particularly salient restorative effect? Third, what are the best strategies for enhancing sleep continuity in maltreated individuals? Fourth, our finding that NVEA was the most important predictor of sleep efficiency (and risk for major depression in males) was unexpected and needs to be verified as this may have important implications for successful parenting.
